# Supplementary material for: Causal Relationship between Aging and Anorexia Nervosa: A White-Matter-Microstructure-Mediated Mendelian Randomization Analysis
Source: Biomedicines. 2024 Aug 16;12(8):1874. doi: 10.3390/biomedicines12081874 (PMC11351342; doi:10.3390/biomedicines12081874)
Supplement: Supplementary file 1 [file biomedicines-12-01874-s001.zip › Supplementary Figure/Figure S4/Singe SNP plot of MR analysis for TL to ubm-b-2029.pdf]

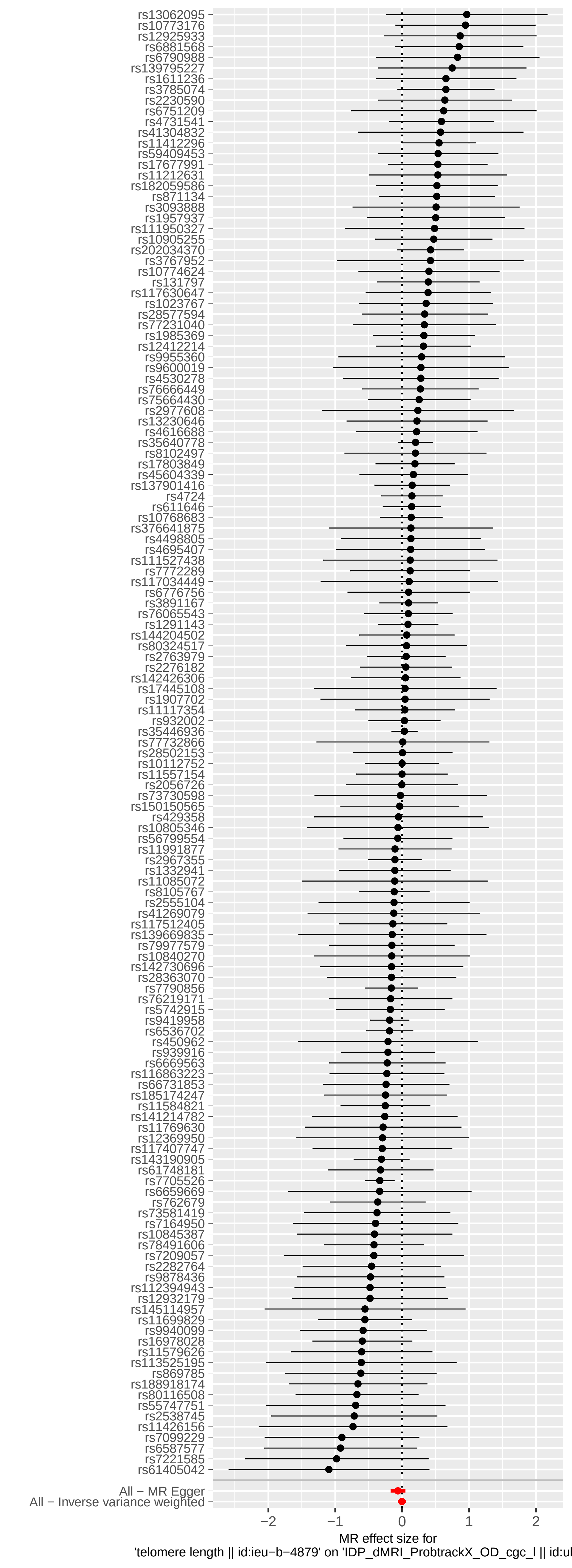
rs13062095
rs10773176
rs12925933
rs6881568
rs6790988
rs139795227
rs1611236
rs3785074
rs2230590
rs6751209
rs4731541
rs41304832
rs11412296
rs59409453
rs17677991
rs11212631
rs182059586
rs871134
rs3093888
rs1957937
rs111950327
rs10905255
rs202034370
rs3767952
rs10774624
rs131797
rs117630647
rs1023767
rs28577594
rs77231040
rs1985369
rs12412214
rs9955360
rs9600019
rs4530278
rs76666449
rs75664430
rs2977608
rs13230646
rs4616688
rs35640778
rs8102497
rs17803849
rs45604339
rs137901416
rs4724
rs611646
rs10768683
rs376641875
rs4498805
rs4695407
rs111527438
rs7772289
rs117034449
rs6776756
rs3891167
rs76065543
rs1291143
rs144204502
rs80324517
rs2763979
rs2276182
rs142426306
rs17445108
rs1907702
rs11117354
rs932002
rs35446936
rs77732866
rs28502153
rs10112752
rs11557154
rs2056726
rs73730598
rs150150565
rs429358
rs10805346
rs56799554
rs11991877
rs2967355
rs1332941
rs11085072
rs8105767
rs2555104
rs41269079
rs117512405
rs139669835
rs79977579
rs10840270
rs142730696
rs28363070
rs7790856
rs76219171
rs5742915
rs9419958
rs6536702
rs450962
rs939916
rs6669563
rs116863223
rs66731853
rs185174247
rs11584821
rs141214782
rs11769630
rs12369950
rs117407747
rs143190905
rs61748181
rs7705526
rs6659669
rs762679
rs73581419
rs7164950
rs10845387
rs78491606
rs7209057
rs2282764
rs9878436
rs112394943
rs12932179
rs145114957
rs11699829
rs9940099
rs16978028
rs11579626
rs113525195
rs869785
rs188918174
rs80116508
rs55747751
rs2538745
rs11426156
rs7099229
rs6587577
rs7221585
rs61405042
All − MR Egger
All − Inverse variance weighted
−2
−1
0
1
2
MR effect size for
'telomere length || id:ieu−b−4879' on 'IDP_dMRI_ProbtrackX_OD_cgc_l || id:ul
